# The effects of an increase in the retirement age on health care costs: evidence from administrative data

**DOI:** 10.1007/s10198-022-01535-w

**Published:** 2022-10-23

**Authors:** Johannes Geyer, Mara Barschkett, Peter Haan, Anna Hammerschmid

**Affiliations:** 1 Department of Public Economics, DIW Berlin, Mohrenstr. 58, 10117 Berlin, Germany; 2 Berlin School of Economics, Berlin, Germany; 3 School of Business and Economics, FU Berlin, 14195 Berlin, Germany

**Keywords:** Pension reform, Health care costs, Difference-in-differences, I10, I12, I18, J14, J18, J26

## Abstract

In this paper, we use unique health record data that cover outpatient care and the associated costs to quantify the health care costs of a sizable increase in the retirement age in Germany. For the identification, we exploit a sizable cohort-specific pension reform which abolished an early retirement program for all women born after 1951. Our results show that health care costs significantly increase by about 2.9% in the age group directly affected by the increase in the retirement age (women aged 60–62). We further show that the cost increase is mainly driven by the following specialist groups: Ophthalmologists, general practitioners (GPs), neurology, orthopedics, and radiology. While the effects are significant and meaningful on the individual level, we show that the increase in health care costs is modest relative to the positive fiscal effects of the pension reform. Specifically, we estimate an aggregate increase in the health costs of about 7.7 million euro for women born in 1952 aged 60–62 which amounts to less than 2% of the overall positive fiscal effects of the pension reform.

## Introduction

Aging populations and demographic change challenge the financial stability of public pension systems. Therefore, many countries reform their pension systems and prolong work lives to increase contributions and to reduce the number of benefit recipients. However, an increasing retirement age might have adverse effects in other areas of the welfare state, specifically for the health care system. Previous studies (e.g. [1, 3, 31, 37]) have documented that a prolonged working life can have negative health effects for individuals.^1^ Yet, so far there exists no clear evidence how these negative health consequences affect health care costs. To assess the overall fiscal effects of pension reforms, this information is crucial.

This paper uses unique data that cover outpatient care and associated costs to quantify the health care costs of a sizable increase in the retirement age.^2^ The data include the universe of individuals insured through the German public health care system (almost 90% of the German population) and comprise a ten-year observation period (2009–2018). In addition to the overall health cost effect of the pension reform, the data also allow us to quantify separate cost effects for different medical specialist categories.

We exploit a sizable cohort-specific pension reform that abolished an early retirement scheme for all women born after 1951. The reform provides a clean quasi-experimental setting as it induces a substantial discontinuity in retirement behavior for two adjacent cohorts. We use this discontinuity in a difference-in-differences (DiD) estimation. This framework accounts for cohort and seasonality effects and allows us to identify the causal effect of the pension reform on health care costs. Specifically, similar to previous studies (e.g. [3, 36]), we define a treatment group (women born between October 1951 and March 1952) and a control group (women born between October 1950 and March 1951) and compare the health care costs of these groups over time.

Our results show that outpatient care costs significantly increase by about 2.9% (about 16 euro per individual) in the age group directly affected by the increase in the retirement age (60–62). Moreover, we also find expectation effects for women at the age of 59 and indirect post-employment effects for women between 63 and 65. We further show that the cost increase is mainly driven by utilization of the following specialist groups: Ophthalmologists, general practitioners (GPs), oral and maxillofacial surgery, neurology, orthopedics, and radiology. The absolute effect is largest for GPs (about 3.5 euro) and thereby contributes about 25% to the increase in the overall costs. While the effects are significant and meaningful on the individual level, we show that the increase in health care costs are modest relative to the positive fiscal effects of the pension reform. Specifically, we estimate an aggregate increase in the health costs of about 7.7 million euro for women aged 60–62 and born in 1952. The corresponding estimate of the net effects of the pension reform for the tax and transfer system including social security amounts to about 4 billion euro [18].

Thus, from an aggregate perspective, our results of an increase in the health care costs do not provide strong evidence against an increase in the retirement age. However, the increase of costs on the individual level shows that positive fiscal effects of a longer working life can be counteracted by potential negative health consequences for individuals. Moreover, our cost estimate focuses on the public health care costs and abstracts from individual disutility or other disadvantages due to worse health as well as other societal costs such as a decrease in labor productivity or an increase in sickness absence at work. For political decisions on retirement ages, such factors also need to be taken into account.

There exists a large literature on the health effects of retirement and pension reforms:^3^ some studies are based on survey data and explore effects of retirement on mental, physical or general health (e.g. [1, 2, 4, 11, 14, 15, 20, 21, 32]). Others use administrative data and consider mortality (e.g. [10, 16, 30]) or health care usage and diagnoses as outcome variables (e.g. [3, 22, 24, 31, 34]). The evidence of this literature is mixed and strongly depends on the pension reform^4^ and the health outcomes^5^ considered. Often broad health measures disguise effects of pension reforms on specific health outcomes. For example, using the same data source, Barschkett et al. [3] show that the reform considered in this study specifically affects mental health, musculoskeletal diseases, and obesity. They find prolonging working life increases the prevalence of all mentioned diagnoses. The underlying reasons for the association between (mental) health and retirement may by mani-folded: different stress-levels in and out of the labor force, changes in social contacts and mobility/movement are some examples.

Despite this sizable literature on health outcomes, there is only little evidence on the effects of pension reforms on public health care costs. Two examples are studies looking at pension reforms that delayed retirement with mixed evidence. Shai [37] finds negative health effects of continued working and an increase in health care consumption in Israel. In contrast, Perdrix [35] shows the opposite effect for France: she finds that later retirement leads to lower health care consumption. Associated with the lower number of doctor visits, she also finds lower expenditure.^6^

Our paper is structured as follows: In “Institutional background” section, we describe the German pension and health care systems. “Data” section provides an overview on the data and “Empirical strategy” section explains the empirical strategy. In “Results” section, we describe the results and compare the additional costs for health care to the overall fiscal effects of the 1999 pension reform. “Conclusion” section concludes.

## Institutional background

In this section, we provide a brief overview on the relevant institutions of the German pension system and discuss the 1999 pension reform, which induced an exogenous increase in the early retirement age for women born after 1951. Moreover, we describe the German health care system.

### Pension system

The German public pension system covers roughly 90% of the workforce.^7^ Pension benefits account for about two-thirds of gross income of the elderly. The system is financed by a pay-as-you-go (PAYG) scheme and has a strong contributory link. The statutory pension age (SRA) was 65 for cohorts born before 1947. It is raised stepwisely to age 67 and fully phased in for all cohorts born in 1964 or later. For the 1951 cohort, the SRA was 65 and 5 months, for those born in 1952 it was 65 and 6 months. People qualify for this regular old-age pension after five years of pension contributions.

Women born before 1952 could retire before the SRA (with permanent deductions) at the age of 60 via the *pension for women*. The 1999 reform abolished this pathway to retirement for cohorts born after 1951. Effectively, the reform raised the early retirement age (ERA) for most women from 60 to 63, which implies an extension of the working life of three years. The eligibility criteria of the *pension for women* were: (i) at least 15 years of pension insurance contributions; and (ii) at least 10 years of pension insurance contributions after the age of 40. About 60% of all women born in 1951 were eligible for the old-age pension for women [17].^8^

### Health care system

German residents are required to have health insurance.^9^ About 90% of the population is insured in the public health care system.^10^ People who opt out of the public system need to insure themselves in a private health insurance plan. Importantly, the insurance status is not affected by entry in retirement. Individuals with a public health insurance during the working life remain in this insurance during retirement.

Public health insurance is financed primarily through mandatory contributions by employers and employees^11^, along with tax revenues. The public insurance offers insurance for non-contributing family members (family insurance). For individuals who receive unemployment benefits, the unemployment agency covers the contributions. For retirees, the pension insurance co-finances the contributions.

In Germany, publicly insured patients do not need to advance the costs of insured health care services. Instead, medical service providers settle their accounts via their regional Association of Statutory Health Insurance Physicians. Price and quantity parameters in the health care system are negotiated on a yearly basis by the National Association of Statutory Health Insurance Physicians and the National Association of Statutory Health Insurance Funds as well as their regional counterparts (see “Appendix A: prices and quantities in the German health care system” section).

## Data

We use administrative data covering the period of 2009 to 2018. The data stem from the database of claims of all publicly insured individuals in Germany as collected by the National Association of Statutory Health Insurance Physicians (KBV). For the analysis we use information on all insured women born between 1950 and 1952.^12^ In addition to the group of women around the cutoff date of the pension reform (women born in late 1951 and in early 1952), we construct a control group consisting of women born late in 1950 and early in 1951.

The data include information for each patient about services and associated costs that medical specialists billed. For each patient the data contain yearly aggregated costs and costs that are specific to medical specialists.^13^ In other words, each patient constitutes an entry for each year in the data set including information about the aggregated costs as well as the specific costs for each of the medical specialists.^14^ The final data set includes about 500,000 women per birth cohort resulting in 1.5 million women overall. While the data includes detailed information on health outcomes and health costs, the data provide no information on important demographic variables such as education, employment status or income. Therefore, we cannot study the heterogeneous costs effects of the pension reform.

## Empirical strategy

We estimate the effect of an increase in the retirement age on health care costs using a DiD estimation strategy. The medical literature (e.g. [8, 13]) documents that month of birth can affect health. Despite the set-up calling for an RDD approach, we prefer the DiD strategy as this allows us to account for seasonality. Specifically, following [36] and [3], we define a control group (women born between October 1950 and March 1951) and a treatment group (women born between October 1951 and March 1952). Women born between January and March are considered to be born after the cutoff. Thus, the interaction term between treatment group and being born after the cutoff estimates the effect of the pension reform. Importantly, the sample only includes individuals born between October 1951 and March 1952 as well as between October 1950 and March 1951, respectively. Thus, birth months between March and October are not included in the sample. This way, we avoid comparing birth months that are rather far away from the reform cutoff in January.

We account for correlation between observations of the same individual or individuals born in the same month, and use robust standard errors clustered by month of birth. In the subgroup analysis (costs by medical specialist), we additionally adjust the standard errors for multiple hypotheses testing using the Bonferroni-correction.

More formally, we estimate the following equation:

1 $$\begin{aligned} y_{it} = \alpha + \beta _0 Cohort_i + \beta _1 Month_i + \beta _2 Cohort_i \times Month_i + Z_{it}\delta + \varepsilon _{it} \end{aligned}$$

where $Cohort_i$ indicates whether individual *i* was born between October 1951 and March 1952. The indicator is zero if individual *i* was born between October 1950 and March 1951. $Month_i$ is the reform indicator that is one if individual *i* was born between January and March and zero otherwise. $Cohort_i \times Month_i$ is the interaction between the two indicator variables and turns one for every woman born from January 1952 on. Thus, the interaction term marks the individuals who are affected by the reform. In addition, we account for age effects captured in $Z_{it}$.

**Fig. 1:**
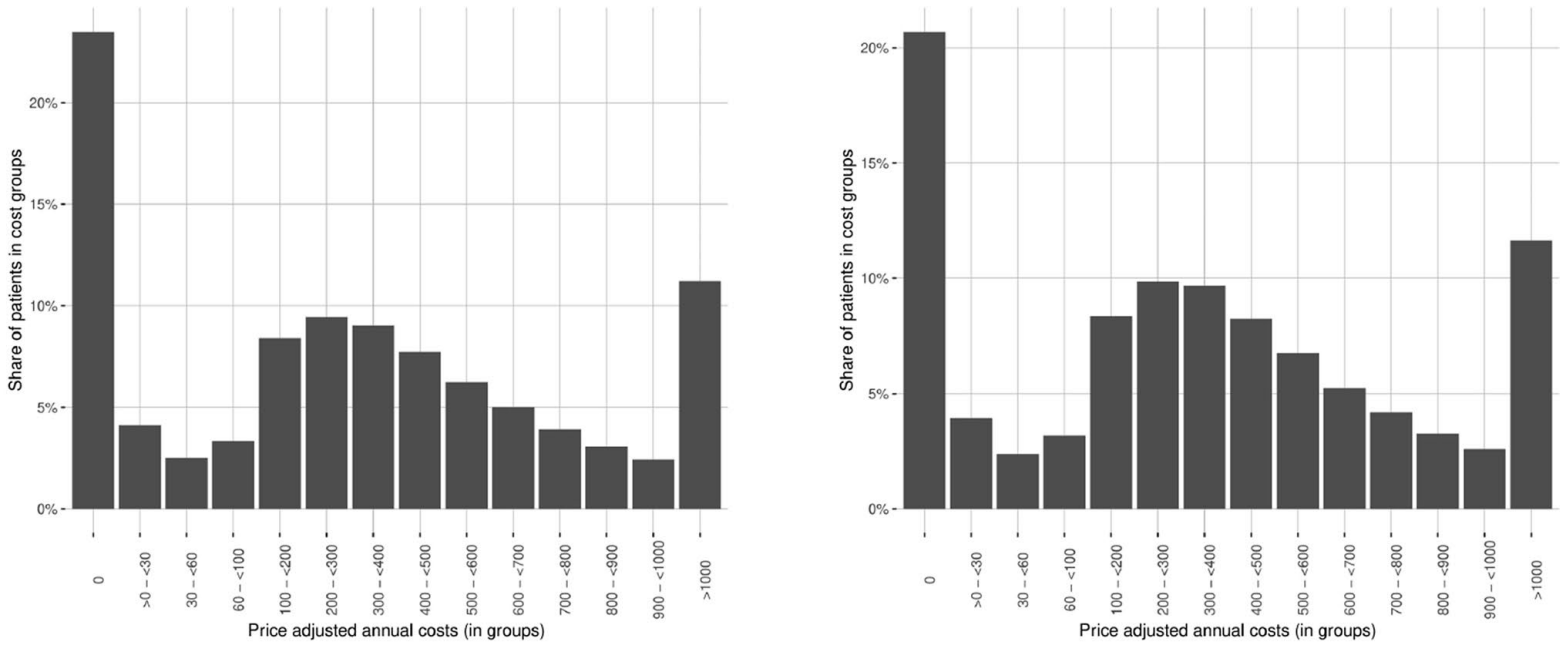
Cost distributions at age 59 and 60 (birth cohort 1951). The left figure presents the costs distribution of women aged 59 years born in 1951. The right figure presents the costs distribution of women aged 60 years born in 1951. Costs are fee-adjusted. Source: KBV, own calculations

The distribution of health costs for the pre-reform cohort (born 1951) at age 59 and 60 (Fig. 1) shows a strong non-linear pattern. While 20–25% of patients produce zero costs per year, about 50% of patients produce between 100 and 600 Euros costs annually. Due to the non-linearity in the aggregated costs variable and the high share of patients with zero cost we estimate in the main analysis two different models and analyze the effects on the extensive margin and the intensive margin. We estimate the extensive margin in a linear probability model (LPM) in which the outcome variable $y_{it}$ indicates if patients produce costs greater than zero in a given year. For the intensive margin we focus only on positive values and define the outcome $y_{it}$ as the logarithm of the total cost. We also estimate the effect of the overall costs including both the intensive and the extensive margin using the linear costs as an outcome variable. When estimating the effect of the reform on specialist-specific costs, we only focus on the linear model and combine the intensive and extensive margin.^15^

To identify a causal effects in a difference-and-difference estimator the standard assumptions need to hold. First, the intervention needs to be unrelated to the outcomes at baseline. Since treatment and control group are determined by birthday this assumption is not problematic in our setting. For the same reason the composition of treatment and control group is stable and there are no spillover effects. Secondly, we provide graphical evidence that the parallel trends assumption holds (parallel trends in the outcomes of treatment and control group prior to the intervention) in the “Appendix C: Robustness” section.

## Results

**Fig. 2:**
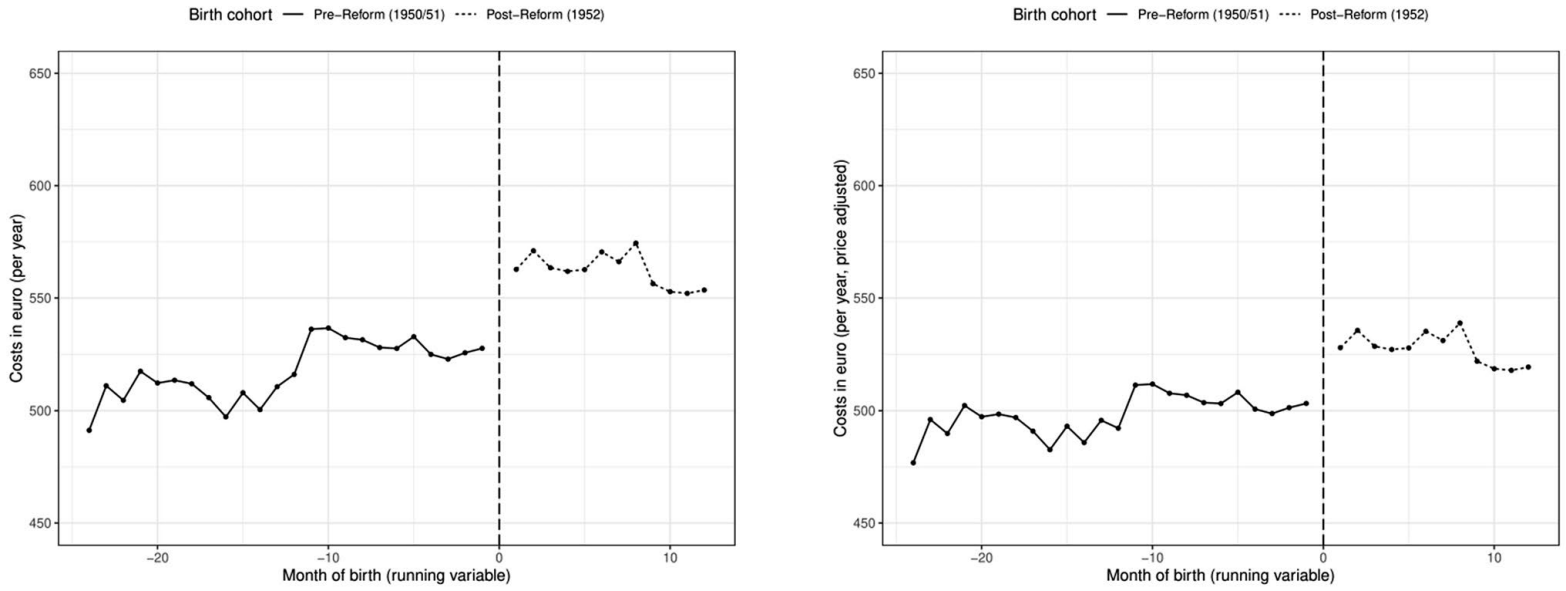
Annual health care costs with and without fee adjustment (1950–52). The left figure presents the average health care costs per year of women between age 60 and 62 for each birth month. The right figure presents the fee-adjusted average health care costs per year (in 2009 fees) of women between age 60 and 62 for each birth month. The vertical lines represent the cutoff date (01/1952). Source: KBV, own calculations

Before we turn to the discussion of the estimation results, we present graphical evidence on the effect of the pension reform on health care costs. Figure 2 shows the average health care costs per year for women aged 60–62 for each birth month.^16^ In the left panel we show the raw data. The right hand side presents the adjusted^17^ health care costs (in fees of year 2009). The vertical lines represent the cutoff date January 1, 1952. For the interpretation, it is important to account for fee changes, since in every year relevant parameters of the health care system are adjusted (see “Appendix A: prices and quantities in the German health care system” section). Therefore, in the fee-adjusted health care costs, the jump between years is smaller. Still, we observe variation in the costs between the months of birth which are related to the seasonality pattern of health (e.g. [8, 13]). In the regression analysis, we account for the fee variation and seasonality using adjusted health care costs and using the DiD framework.

Importantly, at the cut-off, we observe the largest jump in health care costs: the fee-adjusted costs increase by about 25 euro per person after the cut-off date which corresponds to a relative increase of about 5%. This is first evidence that the increase in the retirement age leads to a sizable increase in health care costs. In the following, we turn to the estimation results of the DiD specification to empirically assess this reform effect.

We estimate the effect of the pension reform on health care costs on the intensive and extensive margin for different age groups. In Table 1 we focus on the intensive margin. In the first Column, we present the results for all women aged 59–65. In Column 2, we focus only on women aged 59. Women in this age group were not directly affected by the reform, since retirement via the pension for women was not possible before the age of 60. However, [3] document sizable expectation effects of the 1999 pension reform for several health outcomes, which might affect heath care costs. In Column 3, we consider women aged 60–62. Finally, in Column 4 the results for women aged 63–65 are presented. These results can be interpreted as post-employment effects since women from the treatment and the control group both have the option to retire.

**Table 1:** DiD: price adjusted annual costs (in logs) Source: KBV, own calculations

	Dependent variable: annual costs (in logs)			
	Age: 59–65	Age: 59	Age: 60–62	Age: 63–65
$Cohort_{i} \times Month_{i}$	0.022** (0.008)	0.033** (0.010)	0.029*** (0.008)	0.011 (0.009)
$Cohort_{i}$	0.0003 (0.004)	− 0.009 (0.009)	−0.002 (0.004)	0.006* (0.003)
$Month_{i}$	0.016* (0.007)	0.040*** (0.007)	0.006 (0.007)	0.018* (0.008)
Pre-treatment mean	5.884	5.818	5.866	5.926
Age group included	59–65 years	59 years	60–62 years	63–65 years
Control for age	Yes	No	Yes	Yes
Observations	3,294,970	482,177	1,425,656	1,387,137

Standard errors are clustered on month of birth (running variable) and robust. Column (1) shows the DiD estimates for women aged 59–65 years, column (2) for women aged 59 years, column (3) for women aged 60–62 years and column (4) for women aged 63–65 years. All specifications include age as control variable, except for column (2). All regressions include the cohort indicator, the reform indicator and their interaction term. Costs are fee-adjusted and in logs (zeros are excluded)

$$^{+}$$*p* < 0.1; *p < 0.05; **p < 0.01; ***p < 0.001

The estimation results confirm the graphical evidence: We find that the pension reform, i.e. the shift in the retirement age from 60 to 63, increases health care costs (Table 1). In all specifications (except for 63–65 year old women), the interaction effect that measures the causal effect of the reform, is positive and significant at the 1% or 0.1% level. Specifically, for women aged 59–65 (Column 1), the estimates suggest that the annual health care costs increase on average by about 2.2%. According to the linear specification (Table 3 in the “Appendix B: additional results” section) this corresponds to an increase of about 14 Euros per person. Note, this effect is smaller than suggested by the graphical evidence, which is due to the seasonality pattern that we account for in the DiD estimation.^18^ The effect size over the different age groups is similar. The sizable effect for women aged 59 of over 3% underlines the importance of the expectation effect. At the same time, the insignificant effect on health care costs of women aged 63–65 implies that the pension reform did not lead to persistent increases in health care costs. However, the linear specification (“Appendix B: additional results” section) suggests a significant increase of about 11 Euros for this age group.

In Table 2 we turn to the extensive margin. The results suggest that the increase in healthcare costs can be mostly attributed to increases at the intensive margin. In other words, the additional costs are mainly produced by the group of women with positive costs in absence of the reform. Apart from the age group of 59 year old women, we do not find evidence that the reform induced women to switch from zero healthcare costs to non-zero healthcare costs.

In Fig. 5 we extend the analysis and account directly for the non-linear cost structure documented in Fig. 1. Specifically, we re-estimate the model with 100 different indicator variables for which we increase the threshold in ten euro increments and present the reform coefficients and confidence intervals. The first coefficient is identical to the extensive margin. Overall, the coefficients have a similar magnitude over the cost distribution but at higher costs the point estimates tend to be smaller but in general they are still significant.

**Table 2:** DiD: extensive margin Source: KBV, own calculations

	Dependent variable: annual costs (dummy variable)			
	Age: 59–65	Age: 59	Age: 60–62	Age: 63–65
$Cohort_{i} \times Month_{i}$	0.009$^{+}$ (0.005)	0.019** (0.006)	0.008 (0.006)	0.006$^{+}$ (0.004)
$Cohort_{i}$	0.020*** (0.0004)	0.011*** (0.0004)	0.022*** (0.0003)	0.021*** (0.001)
$Month_{i}$	0.013** (0.004)	0.003 (0.005)	0.014** (0.005)	0.016*** (0.003)
Pre-treatment mean	0.831	0.758	0.808	0.887
Percentage increase in %	1.142	2.558	1.007	0.729
Age group included	59–65 years	59 years	60–62 years	63–65 years
Control for age	Yes	No	Yes	Yes
Observations	3,907,590	627,168	1,737,602	1,542,820

Standard errors are clustered on month of birth (running variable) and robust. Column (1) shows the DiD estimates for women aged 59–65 years, column (2) for women aged 59 years, column (3) for women aged 60–62 years and column (4) for women aged 63–65 years. All specifications include age as control variable, except for column (2). All regressions include the cohort indicator, the reform indicator and their interaction term. The outcome variable is a dummy turning 1 if costs are greater than 0 and zero otherwise

$$^{+}$$*p* < 0.1; *p < 0.05; **p < 0.01; ***p < 0.001

We provide empirical evidence for our identification strategy in “Appendix C: Robustness” section. First, the pre-reform time trends for the treatment and the control groups for the aggregated healthcare costs are very similar (Fig. 6) and, second, the estimates of a placebo test are not significant (Table 6 for the log-specification and Table 7 for the extensive margin). Specifically, for the placebo test we use the same empirical specification but artificially shift the design by one year and assign the cohort born in the first quarter 1951 as the treatment group after the hypothetical reform.

### Results by medical specialist

**Fig. 3:**
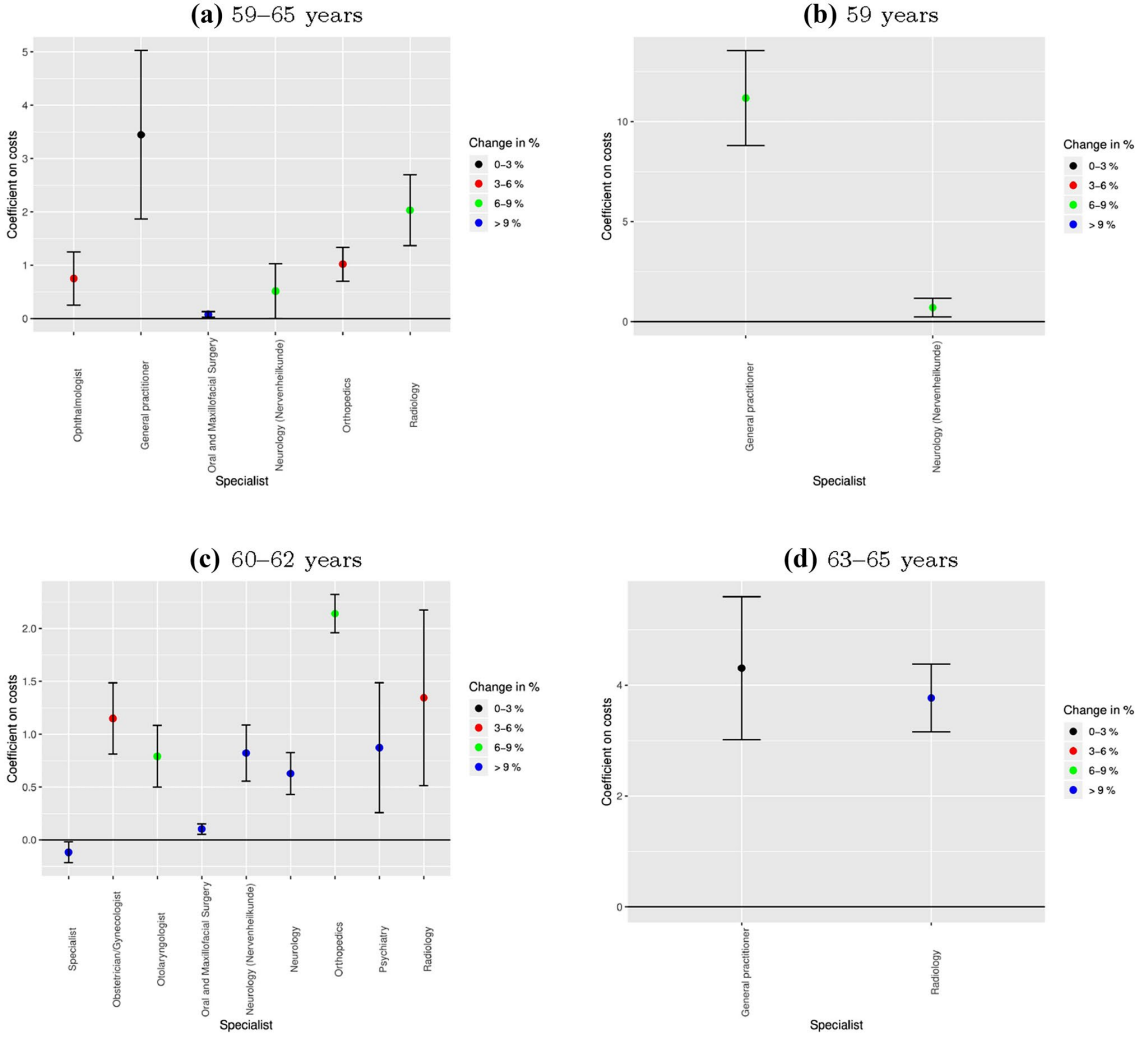
Significant DiD results by medical specialist. There is a new (“Neurologie”) and an old (“Nervenheilkunde”) term for the specialist “Neurology”. Figures show the statistically significant coefficients (with 95% confidence interval) of the DiD regressions on the specialist specific costs. Standard errors are Bonferroni corrected for multiple hypothesis testing. Panel **a** includes estimates for women aged 59–65 years, panel **b** for women aged 59 years, panel **c** for women aged 60–62 years and panel **d** for women aged 63–65 years. Source: KBV, own calculations

**Fig. 5:**
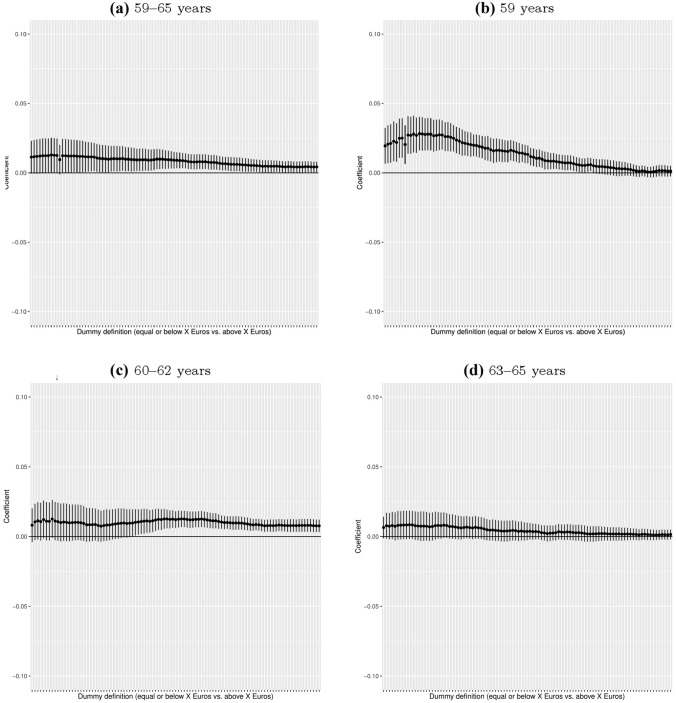
Effects along the cost distribution. Coefficients from estimating 100 times Eq. 1 with different definitions of the outcome variable. In the first regression, the outcome variable is defined as an indicator variable, taking value zero if healthcare costs are zero and one if healthcare costs are greater than zero, i.e. the extensive margin. In the second regression, the indicator is one if costs $\le$ 10 euros and one if costs > 10 euros. In the third regression, the indicator is one if costs $\le$ 20 euros and one if costs > 20 euros. The threshold increases with increments of 10 euros up until 1000 euros. Source: KBV, own calculations

**Fig. 6:**
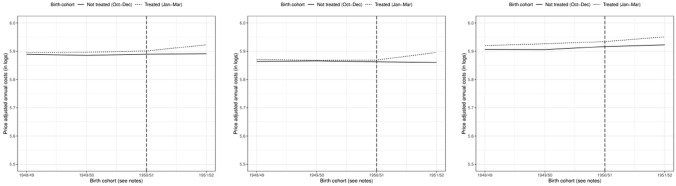
DiD Graphs for 60–65, 60–62 and 63–65 year old women. The left figure presents the average annual costs of women between age 60 and 65 for each birth cohort, the middle figure the costs for women aged 60–62 and the right figure for women aged 63–65 years. The vertical lines represent the cutoff date (01/1952). Birth cohort 1948/49 represents women born between October to December 1948 (control group) and January and March 1949 (treatment group). Accordingly, birth cohorts 1949/50 represent women born between October to December 1949 and January and March 1950, birth cohorts 1949/50 represent women born between October to December 1950 and January and March 1951 and birth cohorts 1951/52 represent women born between October to December 1951 and January and March 1952. Source: KBV, own calculations

In a next step, we analyze to which specialist care utilization the overall increase in costs can be attributed. This analysis provides insights into whether the increased prevalence of certain diagnoses goes along with increased utilization of the relevant specialists. We present the results for health care costs for the 28 different medical specialists that are classified in the data. To correct for multiple hypothesis testing, we adjust the standard errors using a Bonferroni-correction. Figure 3 shows the point estimates and 95% confidence bands for the specialists for whom we find significant effects. Costs are fee-adjusted with the same fee index as the overall costs. The complete results for all specialists can be found in the “Appendix B: additional results” section in Table 4 (general fee adjustment) and Table 5 (adjusted for specialist-specific fees).

Panel (a) shows the results for 59–65 year old women. The increase in the retirement age leads to a significant increase in costs for six specialist groups: Ophthalmologists, GPs, oral and maxillofacial surgery, neurology, orthopedics and radiology. The absolute effect is largest for GPs (about 3.5 euro) and thereby contributes about 25% to the increase in the overall costs. In terms of relative price increases, the effects are largest for oral and maxillofacial surgery, neurology, and radiology.

Panel (b) depicts the results for women at age 59 and the bottom left (Panel (c)) and right panel (Panel (d)) for women aged 60–62 years and 63–65 years, respectively. The results for the 59 year olds suggest, that the increase in the overall costs is mainly driven by increases in the utilization of GP and neurology care. For women aged 60–62 years, we find significant increases in the costs for eight specialists: Obstetricians/gynecologists, otolaryngologist, oral and maxillofacial surgery, neurology, orthopedics, psychiatry, and radiology. In absolute terms, the effects are largest for orthopedics (2.1 euro), radiology (1.3 euro) and obstetricians/gynecologists (1.2 euro). Relatively speaking, the rise in costs for oral and maxillofacial surgery, neurology and psychiatry are largest. The costs for specialists decreases due to the reform by 0.1 euro. Similarly to the 59 year old, the increase in overall costs for the 63–65 year old seems to be driven by increased utilization of mainly two specialist groups: GPs and radiology.

Overall, and across the different age groups the results show a relatively clear pattern. We find the strongest increase in the costs for neurology, psychiatry, radiology, GPs, orthopedics and oral and maxillofacial surgery. Based on the data it is not possible to directly identify the mechanism why the costs in the different categories increase. However, the evidence about the effects of the pension reform on health outcomes [3] allows us to draw indirect conclusions about the mechanisms. [3] document a significant increase in mental health, musculoskeletal diseases, and obesity. Moreover, they find an increase in the number of doctor visits. The increase in mental health can explain the cost effects in neurology and psychiatry which can be related to more frequent doctor visits, diagnostics and treatments. The increase in the costs for orthopedics and radiology are consistent with the finding that musculoskeletal diseases increase. Obesity is often related to mental health and has direct effects on musculoskeletal diseases. Therefore, the increase in obesity is likely to be a driver of the costs effects in the discussed categories. It is difficult to explain the costs effects in oral and maxillofacial surgery based on the mentioned diagnoses. One potential reason for the positive effect of a longer working life on the costs in oral and maxillofacial surgery is that employers may cover part of expensive surgery. The cost effects for the GP can be explained since patients often consult the GP before the specialist.

### Costs and revenues of the pension reform reform

In this section we put our findings into perspective and compare the additional health care costs to the overall fiscal effects of the 1999 pension reform. As shown by [17] the pension reform had a strong negative effect on retirement and a large positive effect on employment as well as on unemployment and inactivity. Specifically retirement rates of affected women decreased by about 25 percentage points. Inactivity and unemployment increased by about ten percentage points, employment by more than 14 percentage points. [18] estimate the related short-term effects on government revenues and expenditures which include changes in income taxation, transfer payments and in the social security system. Focusing only on the 1952 cohort and ages 60–62, the net effect of the reform amounts to four billion euro.

Relative to this sizable net effect, the additional aggregated health care costs are modest. As documented in Table 3 we find an average increase in health expenditures for women aged 60–62 of about 16 euro per year.^19^ The cohort size of women born 1952 is about 540,000. Applying the average cost effect and assuming that about 90% of women are covered by the public health care system (see “Institutional background” section), the overall health care costs related to the pension reform amount to about 7.7 million euro per year in the short run. Thus, relative to the fiscal net effect of four billion euro, the health care costs amount to less than 2%. This cost effect is a lower bound as our data only covers outpatient care. Yet, since the pension reform mainly affected mental health, musculoskeletal diseases, and obesity [3] health care costs related to outpatient care are—at least in the short run—of central importance.

## Conclusion

In this paper, we document that an increase in the retirement age leads to a significant increase in health care costs. To identify the causal effect of the increase in the retirement age, we exploit a cohort-specific pension reform which increased the early retirement age by three years between women born in two adjacent cohorts. The analysis is based on data that include the universe of individuals insured through the German public health care system (almost 90% of the German population) and comprises a ten-year observation period (2009–2018). Our results show that health care costs increase overall by about 2.9% for women in the age group directly affected by the increase in the retirement age (60–62). Moreover, we find expectation effects for women at the age of 59 and indirect post-employment effects for women between 63 and 65. In addition, we show that the cost increase is mainly driven by increased utilization of the following specialist groups: ophthalmologists, general practitioners (GPs), neurology, orthopedics and radiology. The absolute effect is largest for GPs (about 3.5 euro) and thereby contributes about 25% to the increase in the overall costs.

While the effects are significant and meaningful on the individual level, we show that the increase in health care costs are modest relative to the positive fiscal effects of the pension reform. Specifically, we estimate an aggregate increase in the outpatient costs of about 7.7 million for women aged 60-62 and born in 1952. Relative to the corresponding estimate of the net effects of the pension reform of about four billion euro [18] this translates into a relative effect of less than 2%.

Thus, from an aggregate perspective, our results of an increase in the health care costs do not provide strong evidence against an increase in the retirement age. However, the increase of costs on the individual level support the findings of previous studies focusing on individual health outcomes, that positive fiscal effects of a longer working life can be counteracted by potential negative health consequences for individuals. Our cost estimation focuses on the public health care costs and abstracts from individual disutility or other disadvantages due to worse health as well as other societal costs such as a decrease in labor productivity or an increase in sickness absence at work. For political decisions on retirement ages, such non-monetary factors also need to be taken into account.

## Figures and Tables

**Fig. 4:**
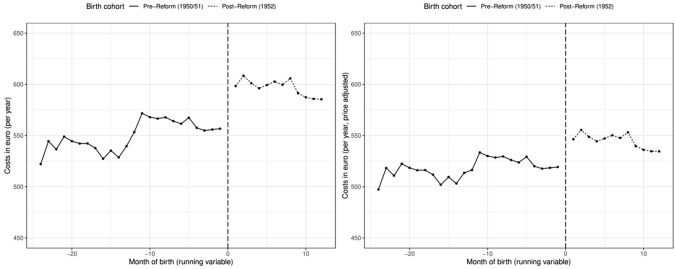
Annual health care costs with and without fee adjustment (1950–52). The left figure presents the average health care costs per year of women between age 59 and 65 for each birth month. The right figure presents the fee adjusted average health care costs per year (in 2009 fees) of women between age 59 and 65 for each birth month. The vertical lines represent the cutoff date (01/1952). Source: KBV, own calculations

## Appendix A: prices and quantities in the German health care system

Every medical service that is covered by public health insurance is valued by a point system (Einheitlicher Bewertungsmaßstab–EBM). Every year, the National Association of Statutory Health Insurance Physicians (KBV) and the National Association of Statutory Health Insurance Funds (GKV Spitzenverband) negotiate at the federal level about the money value of the valuation point (price component) and the morbidity trends (quantity component). Following federal negotiations, the respective regional associations negotiate the specific terms for each region, such as e.g. the regional prices and morbidity parameters that determine the total compensation package.^20^

The total compensation package for outpatient services in each region is financed by the health insurance providers. The respective total compensation packages are split into two parts: the morbidity-related compensation package (MGV) and the extra-budgetary compensation package (EGV).

Medical service providers in the public health care system settle their quarterly accounts with their regional Association of Statutory Health Insurance Physicians (KV) based on the point system^21^ and regional prices. Around 70%^22^ of the medical services are paid from MGV. Since funds in MGV are fixed and limited, medical service providers get paid less than the negotiated rate if they exceed their quarterly ceiling.^23^ Specific services (such as e.g. certain vaccinations) are always covered according to EBM and paid from the EGV budget.

Within the legal framework, the Federal Joint Committee (Gemeinsamer Bundesausschuss; G-BA) decides on questions of coverage of the public health insurance in Germany. This board consists of representatives of public health insurance providers and medical service providers [7].

## Appendix B: additional results

### Graphical evidence (59–65 years)

See Appendix Fig. 4 and Table 3.

**Table 3:** DiD: price adjusted annual costs Source: KBV, own calculations

	Dependent variable: annual costs			
	Age: 59–65	Age: 59	Age: 60–62	Age: 63–65
$Cohort_{i} \times Month_{i}$	14.08* (5.63)	13.87* (5.77)	16.28* (6.57)	11.27* (5.36)
$Cohort_{i}$	9.66*** (2.58)	3.65 (3.29)	9.48*** (2.73)	12.33*** (2.93)
$Month_{i}$	17.55*** (5.14)	20.73*** (5.16)	13.34* (6.14)	21.35*** (4.33)
Pre-treatment mean	517.86	459.30	498.49	563.57
Percentage increase in %	2.72	3.02	3.27	2.00
Age group included	59–65 years	59 years	60–62 years	63–65 years
Control for age	Yes	No	Yes	Yes
Observations	3,907,590	627,168	1,737,602	1,542,820

Standard errors are clustered on month of birth (running variable) and robust. Column (1) shows the DiD estimates for women aged 59–65 years, column (2) for women aged 59 years, column (3) for women aged 60–62 years and column (4) for women aged 63–65 years) All specifications include age as control variable, except for column (2). All regressions include the cohort indicator, the reform indicator and their interaction term. Costs are fee-adjusted

$$^{+}$$*p* < 0.1; **p* < 0.05; ***p* < 0.01; ****p* < 0.001

### Linear specification

#### Effects along the cost distribution

See Appendix Fig. 5.

### Results by medical specialist

See Appendix Tables 4 and 5.

**Table 4:** DiD: annual costs by medical specialist (general fee-adjustment) Source: KBV, own calculations

	Dependent variable: annual costs			
	Age: 59–65	Age: 59	Age: 60–62	Age: 63–65
Anesthesiology	0.20 (0.19)	− 0.27 (0.22)	0.46** (0.17)	0.10 (0.25)
MHT adjusted *p* value	1	1	0.155	1
Pre-treatment mean	7.17	6.75	7.01	7.52
Change in %	2.82	− 4.03	6.55	1.39
Dermatologist	0.06 (0.15)	0.30$^{+}$ (0.18)	0.17 (0.13)	− 0.16 (0.19)
MHT adjusted *p* value	1	1	1	1
Pre-treatment mean	10.69	9.09	10.05	12.05
Change in %	0.56	3.35	1.68	− 1.34
General practitioner	3.45*** (0.70)	11.17*** (1.21)	− 0.10 (0.69)	4.31*** (0.63)
MHT adjusted *p* value	0.000	0.000	1	0.000
Pre-treatment mean	165.01	142.61	161.39	178.17
Change in %	2.09	7.84	− 0.06	2.42
Human genetics	− 0.26* (0.12)	− 0.28 (0.19)	− 0.25 (0.19)	− 0.26 (0.23)
MHT adjusted *p* value	0.923	1	1	1
Pre-treatment mean	1.18	0.83	0.94	1.60
Change in %	− 21.84	− 33.19	− 27.02	− 15.98
Internal medicine	2.77 (2.45)	3.63 (2.50)	2.10 (3.20)	3.18 (2.71)
MHT adjusted *p* value	1	1	1	1
Pre-treatment mean	66.36	55.18	63.01	74.68
Change in %	4.18	6.58	3.33	4.26
Laboratory	0.58** (0.20)	0.80** (0.27)	0.65* (0.27)	0.42 (0.28)
MHT adjusted *p* value	0.113	0.082	0.397	1
Pre-treatment mean	16.51	13.30	15.29	19.18
Change in %	3.54	6.00	4.27	2.19
Medical psychotherapist	0.61 (0.39)	0.12 (0.46)	1.01* (0.43)	0.35 (0.42)
MHT adjusted *p* value	1	1	0.531	1
Pre-treatment mean	4.85	5.45	5.12	4.29
Change in %	12.53	2.19	19.78	8.11
Neurology (Nervenheilkunde)	0.52** (0.17)	0.70*** (0.18)	0.82*** (0.13)	0.09 (0.26)
MHT adjusted *p* value	0.049	0.003	0.000	1
Pre-treatment mean	8.54	8.12	8.47	8.79
Change in %	6.04	8.68	9.70	1.04
Neurology	0.30* (0.13)	0.00 (0.16)	0.63*** (0.10)	0.06 (0.21)
MHT adjusted *p* value	0.498	1	0.000	1
Pre-treatment mean	5.04	4.25	4.50	5.97
Change in %	6	0.04	13.95	0.97
Non-medical psychotherapist	0.54 (0.60)	0.85 (0.83)	0.98 (0.69)	−0.09 (0.65)
MHT adjusted *p* value	1	1	1	1
Pre-treatment mean	13.44	16.24	14.11	11.54
Change in %	3.99	5.22	6.95	-0.81
Nuclear medicine	0.13 (0.09)	−0.14 (0.12)	0.25 (0.16)	0.11 (0.10)
MHT adjusted *p* value	1	1	1	1
Pre-treatment mean	7.00	5.68	6.57	7.90
Change in %	1.88	− 2.41	3.74	1.39
Obstetricians/Gynecologists	0.52** (0.19)	− 0.24 (0.29)	1.15*** (0.16)	0.11 (0.27)
MHT adjusted *p* value	0.189	1	0.000	1
Pre-treatment mean	28.75	29.18	28.22	29.16
Change in %	1.80	− 0.84	4.07	0.39
Ophthalmology	0.75*** (0.16)	− 0.00 (0.23)	1.00** (0.34)	0.78* (0.37)
MHT adjusted *p* value	0.000	1	0.098	0.988
Pre-treatment mean	21.89	13.68	18.54	29.00
Change in %	3.44	− 0.02	5.40	2.68
Oral and Maxillofacial Surgery	0.08*** (0.02)	− 0.06 (0.08)	0.10*** (0.02)	0.11** (0.04)
MHT adjusted *p* value	0.003	1	0.000	0.195
Pre-treatment mean	0.63	0.49	0.58	0.75
Change in %	12.51	− 12.04	17.92	14.41
Orthopedics	1.02*** (0.15)	− 0.59$^{+}$ (0.36)	2.14*** (0.10)	0.41 (0.26)
MHT adjusted *p* value	0.000	1	0.000	1
Pre-treatment mean	29.32	27.47	29.08	30.34
Change in %	3.48	− 2.13	7.36	1.36
Other physicians	0.13 (0.20)	− 1.72 (1.14)	− 0.77$^{+}$ (0.42)	1.90* (0.78)
MHT adjusted *p* value	0.166	0.032	1	0.38
Pre-treatment mean	14.57	10.11	13.41	17.69
Change in %	0.90	− 16.96	− 5.77	10.76
Other service providers	− 0.65 (1.44)	− 0.97 (1.25)	1.37 (1.71)	− 2.79$^{+}$ (1.50)
MHT adjusted *p* value	1	1	1	1
Pre-treatment mean	27.25	25.83	26.79	28.35
Change in %	− 2.37	− 3.74	5.10	− 9.84
Otolaryngologist	0.38** (0.14)	0.07 (0.16)	0.79*** (0.14)	0.03 (0.20)
MHT adjusted *p* value	0.233	1	0.000	1
Pre-treatment mean	10.32	8.90	9.72	11.58
Change in %	3.64	0.75	8.14	0.29
Pathology	− 0.00 (0.04)	− 0.20** (0.07)	− 0.01 (0.09)	0.08 (0.05)
MHT adjusted *p* value	1	0.062	1	1
Pre-treatment mean	5.37	4.63	5.19	5.87
Change in %	− 0.05	− 4.29	− 0.10	1.37
Phoniatrics Pedaudiology	0.00 (0.02)	− 0.00 (0.02)	− 0.01 (0.02)	0.01 (0.02)
MHT adjusted *p* value	1	1	1	1
Pre-treatment mean	0.19	0.09	0.14	0.27
Change in %	0.67	− 3.04	− 4.47	4.18
Physical rehabilitation medicine	0.10$^{+}$ (0.06)	0.13 (0.10)	0.16$^{*}$ (0.08)	0.03 (0.08)
MHT adjusted *p* value	1	1	0.861	1
Pre-treatment mean	2.01	1.76	1.93	2.21
Change in %	5.16	7.19	8.48	1.24
Psychiatry	0.41** (0.15)	− 0.06 (0.21)	0.87*** (0.24)	0.08 (0.10)
MHT adjusted *p* value	0.199	1	0.005	1
Pre-treatment mean	4.67	5.58	4.75	4.22
Change in %	8.77	− 1.09	18.37	1.90
Radiology	2.03*** (0.32)	− 0.32 (0.37)	1.35*** (0.34)	3.77*** (0.32)
MHT adjusted *p* value	0.000	1	0.002	0.000
Pre-treatment mean	28.07	25.40	27.44	29.85
Change in %	7.24	− 1.26	4.90	12.63
Radiotherapy	0.13 (0.20)	− 1.72 (1.14)	− 0.77$^{+}$ (0.42)	1.90* (0.78)
MHT adjusted *p* value	1	1	1	0.38
Pre-treatment mean	14.57	10.11	13.41	17.69
Change in %	0.90	− 16.96	− 5.77	10.76
Specialists	− 0.07* (0.03)	− 0.03 (0.03)	− 0.12*** (0.04)	− 0.04 (0.03)
MHT adjusted *p* value	0.297	1	0.019	1
Pre-treatment mean	0.68	0.76	0.75	0.57
Change in %	− 10.67	− 3.38	− 15.70	− 7.16
Surgery	− 0.04 (0.25)	− 0.31 (0.29)	0.31 (0.37)	− 0.32 (0.22)
MHT adjusted *p* value	1	1	1	1
Pre-treatment mean	16.26	15.07	16.24	16.77
Change in %	− 0.23	− 2.05	1.94	− 1.92
Urology	0.17 (0.17)	0.22* (0.10)	0.27$^{+}$ (0.16)	0.04 (0.23)
MHT adjusted *p* value	1	0.043	1	1
Pre-treatment mean	4.57	4.01	4.26	5.14
Change in %	3.67	5.38	6.23	0.74
Age group included	59–65 years	59 years	60–62 years	63–65 years
Observations	3,904,369	627,097	1,737,117	1,540,155

There is a new (“Neurologie”) and an old (“Nervenheilkunde”) term for the specialist “Neurology”. Standard errors are clustered on month of birth and robust. Column (1) shows the DiD estimates for women aged 59–65 years, column (2) for 59 year old women, column (3) for 60–62 year old women and column (4) for 63–65 year old women. All specifications include age as control variables (except for column (2)). All regressions include the cohort indicator, the reform indicator and their interaction term. Costs are fee-adjusted

$$^{+}$$*p* < 0.1; **p* < 0.05; ***p* < 0.01; ****p* < 0.001

**Table 5:** DiD: annual costs by medical specialist (specific price adjustment) Source: KBV, own calculations

	Dependent variable: annual costs			
	Age: 59–65	Age: 59	Age: 60–62	Age: 63–65
Anesthesiology	0.23 (0.20)	0.00 (0.23)	0.42* (0.18)	0.10 (0.28)
MHT adjusted *p* value	1	1	0.460	1
Pre-treatment mean	7.52	6.75	7.28	8.10
Change in %	3.02	0.06	5.77	1.24
Dermatologist	0.08 (0.14)	0.25 (0.18)	0.18 (0.13)	− 0.11 (0.18)
MHT adjusted *p* value	1	1	1	1
Pre-treatment mean	10.40	9.09	9.81	11.59
Change in %	0.75	2.69	1.85	− 0.92
General practitioner	3.63*** (0.69)	8.75*** (1.20)	0.60 (0.67)	4.97*** (0.61)
MHT adjusted *p* value	0.000	0.000	1	0.000
Pre-treatment mean	161.44	142.61	158.29	172.62
Change in %	2.25	6.14	0.38	2.88
Human genetics	− 0.29* (0.13)	− 0.30 (0.20)	− 0.32 (0.21)	− 0.25 (0.24)
MHT adjusted *p* value	0.660	1	1	1
Pre-treatment mean	1.24	0.83	1.06	1.62
Change in %	− 23.23	− 36.37	− 30.00	− 15.47
Internal medicine	2.94 (2.68)	4.32$^{+}$ (2.58)	2.71 (3.43)	2.63 (3.11)
MHT adjusted *p* value	1	1	1	1
Pre-treatment mean	71.74	55.18	65.83	85.11
Change in %	4.09	7.83	4.11	3.09
Laboratory	0.64** (0.20)	0.45$^{+}$ (0.27)	0.85*** (0.26)	0.49$^{+}$ (0.28)
MHT adjusted *p* value	0.035	1	0.025	1
Pre-treatment mean	16.15	13.30	14.58	19.06
Change in %	3.97	3.41	5.80	2.55
Medical psychotherapist	0.67 (0.43)	0.26 (0.46)	1.05* (0.47)	0.40 (0.47)
MHT adjusted *p* value	1	1	0.707	1
Pre-treatment mean	5.19	5.45	5.44	4.81
Change in %	12.84	4.80	19.36	8.23
Neurology (Nervenheilkunde)	0.53*** (0.15)	0.69*** (0.18)	0.83*** (0.12)	0.12 (0.24)
MHT adjusted *p* value	0.014	0.002	0.000	1
Pre-treatment mean	8.03	8.12	8.00	8.06
Change in %	6.59	8.47	10.45	1.50
Neurology	0.26* (0.12)	− 0.32$^{+}$ (0.17)	0.63*** (0.09)	0.09 (0.20)
MHT adjusted *p* value	0.891	1	0.000	1
Pre-treatment mean	4.95	4.25	4.59	5.65
Change in %	5.30	− 7.52	13.63	1.60
Non-medical psychotherapist	0.53 (0.65)	1.10 (0.87)	0.99 (0.74)	− 0.22 (0.72)
MHT adjusted *p* value	1	1	1	1
Pre-treatment mean	14.33	16.24	14.97	12.85
Change in %	3.72	6.79	6.63	− 1.68
Nuclear medicine	0.12 (0.09)	− 0.23$^{+}$ (0.12)	0.26 (0.17)	0.09 (0.11)
MHT adjusted *p* value	1	1	1	1
Pre-treatment mean	7.36	5.68	6.96	8.50
Change in %	1.58	− 4.00	3.80	1.05
Obstetricians/Gynecologists	0.42* (0.20)	− 0.06 (0.29)	0.75*** (0.17)	0.24 (0.27)
MHT adjusted *p* value	0.862	1	0.000	1
Pre-treatment mean	29.39	29.18	29.22	29.66
Change in %	1.43	− 0.22	2.57	0.82
Ophthalmology	0.69*** (0.16)	0.21 (0.23)	0.74* (0.34)	0.81* (0.34)
MHT adjusted *p* value	0.000	1	0.780	0.439
Pre-treatment mean	21.17	13.68	18.45	27.27
Change in %	3.24	1.56	4.01	2.98
Oral and Maxillofacial Surgery	0.07*** (0.02)	− 0.02 (0.07)	0.09*** (0.02)	0.10** (0.03)
MHT adjusted *p* value	0.001	1	0.000	0.137
Pre-treatment mean	0.57	0.49	0.54	0.65
Change in %	12.79	− 4.36	16.50	14.67
Orthopedics	1.01*** (0.17)	− 0.82* (0.36)	2.20*** (0.10)	0.41 (0.29)
MHT adjusted *p* value	0.000	0.642	0.000	1
Pre-treatment mean	31.19	27.47	30.77	33.16
Change in %	3.23	− 3.00	7.14	1.24
Other physicians	0.23* (0.10)	0.63** (0.20)	0.60** (0.19)	− 0.35** (0.12)
MHT adjusted *p* value	0.616	0.056	0.041	0.071
Pre-treatment mean	7.64	7.42	7.52	7.87
Change in %	3.00	8.43	7.97	− 4.46
Other service providers	− 1.37 (1.34)	− 4.25** (1.45)	0.68 (1.65)	− 2.49* (1.27)
MHT adjusted *p* value	1	0.089	1	1
Pre-treatment mean	26.67	25.83	29.66	23.64
Change in %	− 5.12	− 16.45	2.30	− 10.55
Otolaryngologist	0.36* (0.15)	− 0.04 (0.16)	0.74*** (0.14)	0.10 (0.21)
MHT adjusted *p* value	0.360	1	0.000	1
Pre-treatment mean	10.48	8.90	9.96	11.70
Change in %	3.44	− 0.47	7.43	0.83
Pathology	0.02 (0.04)	− 0.01 (0.06)	0.04 (0.08)	0.01 (0.05)
MHT adjusted *p* value	1	1	1	1
Pre-treatment mean	5.22	4.63	5.01	5.70
Change in %	0.40	− 0.28	0.78	0.25
Phoniatrics pedaudiology	0.00	0.00	− 0.01	0.01
	(0.02)	(0.02)	(0.02)	(0.02)
MHT adjusted *p* value	1	1	1	1
Pre-treatment mean	0.19	0.09	0.14	0.27
Change in %	1.37	0.54	-4.60	4.86
Physical rehabilitation medicine	0.10 (0.06)	0.01 (0.11)	0.20* (0.09)	0.02 (0.09)
MHT adjusted *p* value	1	1	0.526	1
Pre-treatment mean	2.22	1.76	2.11	2.54
Change in %	4.48	0.41	9.56	0.88
Psychiatry	0.43** (0.15)	− 0.06 (0.21)	0.92*** (0.23)	0.09 (0.10)
MHT adjusted *p* value	0.119	1	0.002	1
Pre-treatment mean	4.66	5.58	4.70	4.24
Change in %	9.32	− 1.09	19.47	2.21
Radiology	2.03*** (0.32)	− 0.32 (0.37)	1.35*** (0.34)	3.77*** (0.32)
MHT adjusted *p* value	0.000	0.134	0.000	0.000
Pre-treatment mean	28.07	25.40	27.44	29.85
Change in %	7.24	− 1.26	4.90	12.63
Radiotherapy	0.24 (0.21)	− 0.85 (1.12)	− 0.90* (0.43)	1.97* (0.81)
MHT adjusted *p* value	1	1	0.945	0.385
Pre-treatment mean	14.88	10.11	13.50	18.37
Change in %	1.62	− 8.43	− 6.67	10.75
Specialists	− 0.07* (0.03)	− 0.03 (0.03)	− 0.12*** (0.04)	− 0.04 (0.03)
MHT adjusted *p* value	0.297	1	0.019	1
Pre-treatment mean	0.68	0.76	0.75	0.57
Change in %	− 10.67	− 3.38	− 15.70	− 7.16
Surgery	− 0.06 (0.26)	− 0.13 (0.30)	0.20 (0.38)	− 0.33 (0.24)
MHT adjusted *p* value	1	1	1	1
Pre-treatment mean	0.15 (0.19)	0.09 (0.10)	0.27 (0.18)	0.03 (0.26)
Urology	0.17 (0.17)	0.22* (0.10)	0.27$^{+}$ (0.16)	0.04 (0.23)
MHT adjusted *p* value	1	1	1	1
Pre-treatment mean	4.99	4.01	4.63	5.80
Change in %	2.92	2.18	5.89	0.46
Age group included	59–65 years	59 years	60–62 years	63–65 years
Observations	3,904,369	627,097	1,737,117	1,540,155

There is a new (“Neurologie”) and an old (“Nervenheilkunde”) term for the specialist “Neurology”. Standard errors are clustered on month of birth and robust. Column (1) shows the DiD estimates for women aged 59–65 years, column (2) for 59 year old women, column (3) for 60–62 year old women and column (4) for 63–65 year old women. All specifications include age as control variables (except for column (2)). All regressions include the cohort indicator, the reform indicator and their interaction term. Costs are specialist-specific fee-adjusted

$$^{+}$$*p* < 0.1; **p* < 0.05; ***p* < 0.01; ****p* < 0.001

## Appendix C: Robustness

### Common trends assumption

See Appendix Fig. 6.

### Placebo-tests

See Appendix Tables 6 and 7.

**Table 6:** Placebo-DiD: price adjusted annual costs (in logs) Source: KBV, own calculations

	Dependent variable: annual costs		
	Age: 60–65	Age: 60–62	Age: 63–65
$Cohort_{i} \times Month_{i}$	0.001 (0.009)	0.004 (0.010)	−0.003 (0.010)
Pre-treatment mean	5.893	5.866	5.921
Age group included	60–65 years	60–62 years	63–65 years
Control for age	Yes	Yes	Yes
Observations	2,797,163	1,416,699	1,380,464

Standard errors are clustered on month of birth (running variable) and robust. Column (1) shows the Placebo-DiD estimates for women aged 60–65 years, column (2) for women aged 60–62 years and column (3) for women aged 63–65 years). In the Placebo specification the reform date was artificially shifted to one year earlier (01/1951). All specifications include age as control variable. All regressions include the cohort indicator, the reform indicator and their interaction term. Costs are fee-adjusted and in logs. Zero costs are excluded

$$^{+}$$*p* < 0.1; **p* < 0.05; ***p* < 0.01; ****p* < 0.001

**Table 7:** Placebo-DiD: extensive margin Source: KBV, own calculations

	Dependent variable: annual costs		
	Age: 60–65	Age: 60–62	Age: 63–65
$Cohort_{i} \times Month_{i}$	0.001 (0.005)	0.005 (0.007)	−0.003 (0.003)
Pre-treatment mean	0.894	0.844	0.952
Age group included	60–65 years	60–62 years	63–65 years
Control for age	Yes	Yes	Yes
Observations	3,129,333	1,678,753	1,450,580

Standard errors are clustered on month of birth (running variable) and robust. Column (1) shows the Placebo-DiD estimates for women aged 60–65 years, column (2) for women aged 60–62 years and column (3) for women aged 63–65 years). In the Placebo specification the reform date was artificially shifted to one year earlier (01/1951). All specifications include age as control variable. All regressions include the cohort indicator, the reform indicator and their interaction term. The outcome variable is a dummy turning 1 if costs are greater than 0 and zero otherwise

$$^{+}$$*p* < 0.1; **p* < 0.05; ***p* < 0.01; ****p* < 0.001

## Footnotes

1 There is also evidence for positive effects of retirement on health (e.g. [1, 2, 4, 11, 12, 14, 20, 21, 32]), yet findings are ambiguous and very much depend on the setting under study (e.g. pension reform, healthcare system etc.).

2 Since the pension reform we consider mainly affected mental health, musculoskeletal diseases, and obesity health care costs related to outpatient care are—at least in the short run—of central importance [3].

3 For a more detailed discussion, see e.g. [3].

4 The majority of previous studies on the link between health and retirement use age discontinuities in the retirement age to instrument the individual’s retirement status (see Picchio et al. [38] for an overview of methodologies of previous studies). Only a few studies exploit direct variation from pension reforms (e.g., [5, 11, 15, 21, 30]).

5 Health outcomes differ very much and range from self-assessed general health status to more specific self-assessed outcomes (e.g. cognitive abilities, mobility limitations, grip strength, hypertension, migraine, back pain) to mortality and specific diagnoses assessed by healthcare professionals (e.g. mental disorders, musculoskeletal diseases, cardiovascular diseases, obesity). Due to the wide range of outcomes as well as different assessment methods it is difficult to compare the effects and draw conclusions.

6 Zhang et al. [40] focus on private health expenditures and find for China that retirement increases health care utilization and yearly out of pocket expenditures for inpatient care as well as monthly out of pocket expenditures for self-treatment. For men, they also depict an increase in out-of-pocket inpatient costs.

7 There are a few exemptions from compulsory insurance: civil servants have a separate tax-financed, non-contributory defined benefit scheme and most of the self-employed are not compulsorily insured (for a general description of the German pension system and the pension reform, see [3, 9]).

8 Previous studies evaluate the labor market effects of the 1999 pension reform [17, 18]. Based on data of the pension insurance [17] document that the labor market outcomes are very similar in the treatment and the control group before the age of 60. Moreover, they show that employment rates increased by about 15 percentage points (pre-reform mean 54%) and inactivity and unemployment increased by about 11 percentage points (pre-reform mean 12%). Further they point out that the reform caused women to stay longer in the current status, i.e. employed women continue working, unemployed women stay unemployed and inactive women remain inactive until reaching the new early retirement age. Thus, the negative health effects found by [3] are mostly driven by women who stay longer in employment (e.g. due to increased stress-levels when working compared to being retired) and women who stay longer in unemployment (e.g. lower life-satisfaction due to delayed change of identity from unemployed to retired [23]). In our data we are not able to differentiate the employment status of the women.

9 For most information on the health care system in this section and additional details: [7].

10 There are a few exemptions from compulsory public insurance: e.g. people with an income above a certain threshold (5213 euro monthly earnings in 2020), self-employed, and civil servants, are allowed to opt out of the public insurance.

11 The overall contribution rate in 2020 was 14.6% of gross labor earnings, equally shared by employees and employers.

12 We focus only on cohorts 1950–1952 since a major school reform affected many women born after 1952. Specifically, regional school reforms in West Germany raised compulsory schooling from 8 to 9 years. Four large West German federal states changed compulsory schooling within cohort 1953. The reform had positive effects on health outcomes [29].

13 These costs are reported on the calendar quarter level in the original data. We aggregate the costs specific to medical specialists to the year level. Specialists not relevant for our research question (e.g. pediatricians) are not considered in this analysis.

14 The sample is unbalanced as patients only appear if they received outpatient care at least once per year. Based on this information, we construct a balanced sample with yearly information on all publicly insured individuals. Costs for years without outpatient care are assumed to be zero.

15 Estimates of the intensive margin would not be comparable across specialists as the share with positive values strongly varies.

16 In “Appendix B: additional results” section Fig. 4, we show the same figures for women in the age range 59–65.

17 Fees are adjusted to the year 2009 fees. This adjustment accounts for the general increase in the fee level and specific changes to the medical system (The time series “Honorarumsatz je Behandlungsfall in euro” from 2009–2018 was used to adjust fees [28]). For more information on annual changes in the health care system, see “Appendix A: prices and quantities in the German health care system” section.

18 The positive and significant coefficients of the “Month” indicator are in line with the seasonality pattern found by [3]. It suggests that women born in the first quarter of the year produce higher healthcare costs than women born in the last quarter of the year.

19 This estimate needs to be interpreted as an intent to treat effect (ITT) since not all women were eligible for the pension for women. According to [17] about 60% of women in the cohorts considered were affected by the pension reform reform.
